# Lachesis and Sepia Cases

**Published:** 1892-10

**Authors:** P. C. Sanderson

**Affiliations:** Philadelphia, Pa.


### LACHESIS AND SEPIA CASES.

P. C. Sanderson, M. D., Philadelphia, Pa.

Lachesis$^{4C}$.—I received a call by messenger boy to come at once to a prominent hotel. On arriving there, I found a young woman suffering with the following symptoms: Left side of face badly swollen, nearly closing the eye; purplish look. Also upon the left superior maxillary a large gum-boil; choky feeling in her throat. She feared it would close up and she would die. An old-school physician had seen the case and said to call a dentist and have a recently-filled tooth removed. But she said "No," and sent for me. I gave one dose Lachesis$^{4C}$. She received relief in a few minutes. The boil broke the next morning, the tooth was saved, and she is thankful. So am I—for the patients she sends me.

Sepia$^{6X}$.—A working woman from hotel called at my office for the treatment of the following: Two large wine-colored spots on each cheek. No inflammation, but looked very bad. Was under old-school treatment with salves, etc. I learnt some few years ago while reading The Homœopathic Physician that Sepia would cure such spots. So I gave it, and a cure was the result in about ten days.

